# Editorial: Bioengineering systems for therapeutic and *in vitro* platforms

**DOI:** 10.3389/fbioe.2022.1090317

**Published:** 2022-11-22

**Authors:** Loredana De Bartolo, Antonella Piscioneri, Dimitrios Stamatialis, Feng-Huei Lin

**Affiliations:** ^1^ National Research Council of Italy, Institute on Membrane Technology, CNR-ITM, Rende, Italy; ^2^ Advanced Organ Bioengineering and Therapeutics-Technical Medical Center, University of Twente, Enschede, Netherlands; ^3^ Department of Nephrology- Radboud University Medical Center, Radboud Institute for Molecular Life Sciences, Nijmegen, Netherlands; ^4^ National Health Research Institute of Biomedical Engineering and Nanomedicine, Zhunan, Taiwan

**Keywords:** biomimetic interfaces, functional biomaterials, tissue repair, *in vit*ro tools, microflluidic devices, disease modelling, drug testing, bioreactor

The works presented in this editorial provide a wide and multidisciplinary overview of the latest strategies for the realization of bioengineered systems for therapeutic and *in vitro* platforms. These platforms must ensure that the tissue complexity is recapitulated and maintained to provide reliable novel scientific outcomes. The Research Topic covers various strategies for creating permissive environments, where cells can organize according to a proper architecture (Baptista et al., 2021). The use and the realization of alternative cell culture strategies also allows the fine tuning of operational parameters which offers the possibility to mimic tissue physiology and the appropriate cell niche. To accomplish this fundamental task different approaches are applied. The employment of bioactive material promotes cell material interaction at the interface thus boosting the cellular response in terms of adhesion, proliferation and differentiation. Surface functionalization enhances the performance of the tissue engineered construct by tailoring the biomaterial surface with key functional groups and moieties whose presence recapitulate the physiological surrounding. Other approaches involve the development of dynamic systems as bioreactor or chip devices (Piscioneri et al., 2018). The dynamic cell culture microenvironments realized there enhance nutrients transport and waste removal ensuring at the same time a proper supply of gases. The precise set up of these parameters ensure that cells are grown in a very controlled and optimized microenvironment (Morelli et al., 2019). Moreover, dynamic *in vitro* platform devices are quite versatile systems; indeed, their flexible control of different operational setting pave their use for the replication of cellular and extracellular features of different organs and tissue. The versatility of these multifunctional devices allows the expansion of cell types and therefore the investigation of a wide range of tissue districts, disclosing new insight related to physiological and/or pathological conditions (Morelli et al., 2021). On the basis of these main features these tools can be broadly used for several applications which include the promotion of tissue repair, *in vitro* diseases modelling and preclinical drug screening of potential therapeutic compounds.

The Research Topic summarizes the latest advances in the field of biomimetic therapeutic material and *in vitro* multifunctional platforms that offer an optimal surrounding for cell growth and tissue reconstruction as investigational tools. Original Research articles and case reports from leading experts in their fields highlight the latest exciting achievements referring to the development and application of multifunctional biomaterials and devices in tissue engineering and regenerative medicine.

Recent advances in 3D printing technology have allowed to develop biocompatible materials to be used for repair of tissues and organs as well as for the creation of *in vitro* cellular platforms to establish disease pathogenesis and drug screening model. In this context, Cui et al. developed 3D-printed polylactide cold jackets for laparoscopic complete intracorporeal renal auto transplantation to preserve the renal function of severe renal artery stenosis patients. They presented the first successful application of this concept in the treatment of renal artery stenosis.

A 3D co-culture environment to mimic pathological characteristics of rheumatoid arthritis pannus tissue has been created by Lin et al. This system, based on 3D scaffold constructed by bioprinting technology with synovial fibroblasts, vascular endothelial cells and gelatin/alginate hydrogels, could be suitable for high-throughput drug screening *in vitro* model to evaluate drug efficacy and safety.

The clinical translation of several therapeutic approaches that are at the forefront of scientific and technological innovation, is hindered by the poor predictive capacity of the currently available *in vitro* pathophysiological models. Coentro et al., established an *in vitro* model of skin fibrosis for testing the capacity to decrease collagen synthesis and/or deposition of anti-fibrotic molecules with different mechanisms of action. This study advocated the use of macromolecular crowding and TGFβ 1 in the development of skin fibrosis specific *in vitro* models.

Dynamic devices such as bioreactors show promise in tissue engineering, since they are able to recapitulate the *in vivo* physiological cell environment thanks to a continuous perfusion of gases and nutrients while removing waste products, ensuring the homeostasis of tissue. The study of Yamada et al., emphasized the necessity of optimization of a custom-designed perfusion bioreactor for bone tissue engineering taking into account the key experimental variables to address issues common to perfusion bioreactors for bone tissue engineering.

Within this framework, Ene-Iordache et al. developed a 3D nichoid microenvironment within a miniaturized optically accessible bioreactor housing. There, the combination of the nichoids with the millifluidic bioreactor allows to culture 3D organoids of few millimeters in size under continuous perfusion of the culture medium.

Kumar et al., developed a microfluidic alveolus model to study lung biomechanics. This system consisted of pneumatic and fluidic chambers separated by a thin membrane that supports alveolar epithelial cell culture. The device mimicked strain heterogeneity experienced during alveolar expansion due to breathing and it could serve as a tool to delineate the role of alveolar micromechanics in physiological and pathological outcomes in the lung.

Microfluidic devices are suitable to investigate complex constructs with accurate and controllable environment by high resolution spectroscopies and real-time imaging. A microfluidic platform compatible with advanced widefield- and confocal microscopy was developed to recapitulate an *in vitro* intestinal epithelial barrier for investigating the cellular uptake and cross-barrier transport of biologics (Weller et al.). The microfluidic platform was validated as a suitable model for intestinal drug transport studies by correlating the transport of small molecule drugs to the corresponding human absorption data.

In the context of *in vitro* models, a bioengineered 3D platform based on human intrahepatic cholangiocyte organoids cultured onto polyethersulfone hollow fiber membrane replicated the biological structure and function of native bile ducts (Wang et al.). This platform may be suitable for studying cholangiopathies and therapeutic strategies.

Marzagalli et al. presented an organ-on-chip (OOC)-based approach for recapitulating the immune cell migration under physiological fluid flow. This model was used to study the infiltration within a 3D tumour matrix, and activation against neuroblastoma cancer cells in a fully-humanized, fluid-dynamic, and clinically relevant-sized environment.

In the last years, organoid models using pluripotent stem cells have been implemented for diagnostic and therapeutic utilities. Kakni et al., developed organoids consisting of a simple columnar epithelium patterned into crypt-like and villus-like structures. These organoids reflecting the structural and functional characteristics of the *in vivo* counterparts, could represent a powerful new tool for studies related to diseases, microbiota nutrient absorption and drugs.

In tissue engineering, the impact of the cell type on the degradation of biomaterials has been studied by Boucard et al. They investigated the capacity of primary and immortalized fibroblasts of distinct origins to degrade a fibrin-based biomaterial establishing that fibrin degradation through the secretion of serine protease.

Multifunctional platforms that perform diagnostic functions and serve as responsive biomaterials to tumour microenvironment are encouraging. Sivasubramanian et al., developed a hybrid nanoplatform that harnesses the tumour microenvironment factors for dual targeted magnetic resonance imaging and assessment of tumour oxygen status by photoacoustic imaging. They reported the fabrication of a novel enzyme responsive T1 magnetic resonance imaging contrast agent that can modulate oxygen in the tumour microenvironment *via* the catalytic conversion of H_2_O_2_ to O_2_.

Peripheral nerve transection is one of the most common peripheral nerve injuries. Although neurons have a limited ability to regenerate, axons have a great potential for regeneration. To find an effective treatment strategy for sciatic nerve injury, Lin et al., explored the protective effect of GDF11 on neurons *in vitro*, and a lentiviral vector to induce GDF11 overexpression with the aim to create and maintain a local microenvironment conducive to nerve regeneration.

In the tissue engineering scenario decellularized matrices are used as natural scaffolds to promote the differentiation of implant cells and tissue remodelling. Owing to the removal of immunogenic components, the decellularized matrix has good biocompatibility and safety, which can be used in autologous, allogeneic, and xenogeneic tissue engineering. Tang et al., investigated the effects of tissue origins on the adipogenic capacity of the decellularized matrix exploring the mechanisms that decellularized adipose-derived matrix promotes adipose tissue formation and provides further insight into tissue specificity, which could be applied in different tissue repair and regeneration.

Finally, Kuan et al., synthesized a sonosensitizer of Eu-doped CaCO_3_ to combine with low-intensity ultrasound as a non-invasive treatment for the body sculpture for body sculpture. The CaCO_3_: Eu had good biocompatibility and could produce ROS in adipocytes for lipolysis. The results showed that developed sonosensitizer could effectively inhibit the adipogenesis, after treated with low-intensity ultrasound, without skin burning and charred sounding tissue.
